# Validation of a Pregnancy‐Associated Glycoprotein–Based Lateral Flow Assay for Early Pregnancy Detection in Goats

**DOI:** 10.1002/vms3.71022

**Published:** 2026-06-09

**Authors:** Mehmet Can Kara, Ayşe Merve Köse

**Affiliations:** ^1^ Republic of Turkey Ministry of Agriculture and Forestry, Samandağ District Directorate of Agriculture and Forestry, Samandağ Hatay Türkiye; ^2^ Department of Obstetrics and Gynecology, Faculty of Veterinary Medicine Hatay Mustafa Kemal University Hatay Türkiye

**Keywords:** early pregnancy diagnosis, goat, OnFarm pregnancy test, pregnancy‐associated glycoproteins, progesterone

## Abstract

**Background:**

The significant cross‐reactivity between bovine, ovine, and caprine pregnancy‐associated glycoproteins (PAGs) enables the adaptation of bovine‐specific diagnostics for use in other ruminants; consequently, the Alertys OnFarm Pregnancy Test (AOFPT)—a blood‐based lateral flow assay—provides a rapid and practical solution for pregnancy detection directly at the animal's head under field conditions.

**Objectives:**

This study evaluated to assess and validate the performance of AOFPT in goats at days 21 and 28 post‐mating, by comparing the results with serum progesterone (P4) analysis and using transabdominal ultrasonography (TAUS) as the gold standard.

**Methods:**

The study involved 85 Kilis goats, five months post‐partum. Estrus was synchronized using an 11‐day progestagen device, d‐cloprostenol, and PMSG. Whole blood and serum samples were collected on Days 21 and 28 post‐mating. AOFPT was performed on‐farm immediately after collection. For validation, serum progesterone concentrations were measured via electrochemiluminescence immunoassay, and TAUS was performed on Days 35 and 42, with Day 42 findings serving as the gold standard.

**Results:**

Results indicated that on Day 21, AOFPT sensitivity, specificity, positive predictive value, and negative predictive value were 79.4%, 93.8%, 98.2%, and 51.7%, respectively. By Day 28, these metrics reached 100%, 81.3%, 95.8%, and 100%. Statistical agreement between AOFPT and the reference method was K = 0.55 (82.14%) on Day 21 and K = 0.87 (96.43%) on Day 28 (*p* <0.001). Median ± interquartile range (IQR) of P4 concentrations were 5.95±2.60 ng/mL on day 21 and 6.92 ± 2.93 ng/mL on Day 28. AOFPT‐identified pregnant goats exhibited significantly higher P4 levels (*p* < 0.001) than non‐pregnant goats on Day 21 (5.98 ± 3.21 vs. 4.05 ± 5.46) and Day 28 (6.84 ± 3.06 vs. 0.75 ± 5.96).

**Conclusions:**

AOFPT demonstrated accuracy and reliability closely matching P4 measurements and the reference method. This test provides a practical tool for early on‐farm pregnancy diagnosis in goats, potentially enhancing reproductive management and productivity in dairy goat farms and large herds.

## Graphical abstract

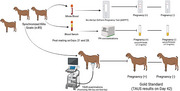

## 1 Introduction

Goat farming profitability depends on both milk and meat production, which is directly linked to reproductive efficiency. Selecting appropriate breeding and herd management strategies to improve reproductive performance—and thereby increase both the quantity and quality of animals—relies on early pregnancy diagnosis (Barbato et al. 2022; Khanum et al. 2008; Zamfirescu et al. 2011). A reliable and appropriate diagnostic method enables timely re‐breeding, culling, or selective management of non‐pregnant animals (Karadaev 2015) while facilitating appropriate nutritional protocols for pregnant does (El Amiri et al. 2015; Sharma et al. 2020b). Additionally, the widespread adoption of controlled breeding techniques, such as artificial insemination and out‐of‐season breeding, in large goat herds has increased the demand for an accurate, practical test for early pregnancy diagnosis (Salve et al. 2016). Pregnancy diagnostic methods may be categorized as direct/indirect or functionally grouped into: (i) visual assessment, (ii) clinical examination, and (iii) laboratory testing (Barık and Yadav 2022; Jainudeen and Hafez 2000; Purohit 2010). While some field‐applicable methods lack sufficient accuracy, others demonstrate high precision but require specialized equipment and technical expertise (Karadaev 2015). The most commonly used methods include hormonal assays, analysis of pregnancy‐associated proteins, and ultrasonography (Karen et al. 2001).

Pregnancy‐associated glycoproteins (PAGs) are a type of inactive aspartic protease that is produced exclusively by the placenta in ruminants (Balhara et al. 2013; El Amiri et al. 2015; Kaya et al. 2016). After secretion by trophoectodermal cells of the placenta, PAGs migrate from foetal tissues and fuse with maternal uterine epithelial cells to form hybrid foeto‐maternal trinucleate cells (Cruz et al. 2024). PAGs become detectable in the peripheral blood of a pregnant animal around the time when the extraembryonic membranes attach to the endometrium, coinciding with the migration of binucleate trophoblast cells to form the embryo–maternal syncytium and their fusion with endometrial cells (Wooding 1984; Younis and Aboud 2023). Therefore, PAGs serve as reliable biomarkers for both pregnancy status and fetoplacental function (Barbato et al. 2022; Garbayo et al. 2000; Sharma et al. 2020b). Blood‐based assays for maternal serum PAGs have become more prevalent in recent years. It is stated that cross‐reactivity may occur due to N‐glycosylation similarities in PAG proteins isolated from cattle, sheep and goats (Garbayo et al. 1998; Sousa et al. 2006). So, commercial PAG tests use antibodies raised against bovine PAGs; however, due to structural similarities across ruminant PAG isoforms, cross‐reactivity occurs between bovine, ovine, and caprine PAGs. This cross‐reactivity enables the use of commercially available bovine tests for pregnancy detection in small ruminants and other ruminant species (Roberts 2022).

The commercial IDEXX Rapid Visual Pregnancy Test (RVPT), which is the test based on enzyme‐linked immunoassay (ELISA) that can be run without ELISA instrumentation and read visually, on the principle of PAG detection, has been evaluated extensively in cattle (Akköse et al. 2025; Mayo et al. 2016; Rashmi et al. 2020; Rice et al. 2013) and sheep (Akköse 2020; Chaves et al. 2017; Chaves et al. 2020; Roberts et al. 2019; Steckeler et al. 2018). In addition, studies using the Alertys OnFarm Pregnancy Test (AOFPT), which is a blood based lateral‐flow test that gives results in 5–20 min—employed in this study—in cattle were conducted by Akköse (2023), Szelenyi et al. (2024), Kline et al. (2024) and Safak et al. (2025). However, except for the investigations by Kim et al. (2020) and Akkaya Doğan and Köse (2022) used RVPT, no studies have evaluated commercially available blood based lateral‐flow test, in goats. Compared to AOFPT, RVPT requires laboratory use (Akkaya Doğan and Köse 2022), while AOFPT, which operates on a blood‐based lateral flow principle and provides rapid results, can be easily used at the animal's head under field conditions (Akköse 2023). Because RVPT analysis results are visually assessed by colour change, it is a test open to subjective evaluation (Akkaya Doğan and Köse 2022).

Therefore, the aim of the present study was to assess and validate the performance of AOFPT—originally designed to detect PAG molecules in bovine serum—in goats at Days 21 and 28 post‐mating, by comparing the results with serum progesterone (P4) analysis and using transabdominal ultrasonography (TAUS) as the gold standard.

## 2 Materials and Methods

### 2.1 Animals and Synchronization Protocol

The animals for this study comprised 85 clinically healthy, fertile Kilis does on post‐partum 150 days and aged 3–5 years, with body weights ranging from 45 to 65 kg (55.17 ± 0.47; mean ± SEM), and 10 bucks with body weights ranging from 55 to 76 kg (67.41 ± 2.12; mean ± SEM), maintained by a producer of the Yayladağı district, Hatay. Animals were turned out to graze on dry forage and crop stubble from 04:00 to 09:00 h and from 16:00 to 20:00 h. After 20:00 h, they were returned to their pens and fed a ration consisting of a mixture of barley grain and hay. Drinking water was provided from both the municipal supply and a clean well source. Estrus synchronization in does was performed during the breeding season (August–September) using an 11‐day intravaginal sponge (Esponjavet, HIPRA, Spain). On the day of sponge removal, an intramuscular injection of 125 µg d‐cloprostenol (Gestavet Prost, HIPRA, Spain) and 500 IU PMSG (Oviser 500, HIPRA, Spain) was administered (Akkaya Doğan and Köse 2022). Twenty‐four hours after sponge removal, bucks were introduced to the flock for 12 h, remaining with the does for 2‐h intervals; thereafter, the bucks were removed. Does exhibiting estrus were mated by hand. Following completion of matings, does were housed in a separate pen.

### 2.2 Blood Sample Collection

Following mating, on Days 21 (between 17:00 and 19:00) and 28 (between 17:00 and 19:00), the blood samples were collected from the jugular vein of each doe using a double‐ended needle (21 G × 1.5 inch; 0.8 × 38 mm) and a plastic vacutainer holder. Samples were drawn into vacuum tubes with gel (no anticoagulant) and into EDTA‐coated tubes (with anticoagulant). Each animal's ear tag number was recorded on the tube. Anticoagulated blood samples were used for AOFPT. Blood samples with no anticoagulant were centrifuged at 3000 rpm for 10 min at room temperature in the laboratory, and blood serum was separated. The separated serum was transferred into labelled Eppendorf tubes and stored at −20°C until P4 assay.

### 2.3 Whole Blood Pregnancy Test Procedure

The Alertys OnFarm Pregnancy Test was conducted on‐farm using whole blood samples without prior knowledge of each animal's pregnancy status, following the manufacturer's instructions (IDEXX, Westbrook, Maine, ABD). The EDTA tube was gently inverted once or twice, and the blood sample and wash solution were sequentially applied to the test well. After a 20‐min incubation, the presence of both the control (C) and test (T) lines indicated pregnancy, whereas appearance of only the C line indicated nonpregnancy.

### 2.4 P4 Measurement

Serum P4 concentrations were measured in the laboratory using an electrochemiluminescence immunoassay (ECL) on a fully automated analyser (Cobas E601, Roche, Switzerland). Does with serum P4 levels of ≥1.5 ng/mL on Day 21 were considered pregnant (Boscos et al. 2003).

### 2.5 Pregnancy Diagnosis by Taus

Pregnancy examinations were performed twice: first at Day 35 and again at Day 42 post‐mating. All TAUS examinations were conducted by the same clinician using a real‐time ultrasound machine equipped with a 5 MHz convex probe (Falco, Pie Medical, The Netherlands). With the goat standing, the hair over the right paralumbar fossa ventral to the udder was clipped to serve as the scanning window (Dinç et al. 1994). Positive diagnosis of pregnancy by TAUS on Day 35 is assured by imaging the embryo/foetus and foetal heartbeat surrounded by fluid (Dawson 2002; Gonzalez et al. 2004). A positive pregnancy diagnosis was recorded when a fluid‐filled uterus, placentomes, moving foetus, and foetal heartbeat were visualized. These findings were recorded, and the day 42 TAUS result was designated as the gold standard (Wojtasiak et al. 2020).

### 2.6 Statistical Analysis

Descriptive statistics (count, percentage, mean, standard error of means, etc.) were calculated for the recorded variables. Agreement between the Day 21 and Day 28 pregnancy test results and the reference TAUS findings on Day 42 was assessed using Cohen's kappa coefficient. And none (0.0–0.20), minimal (0.21–0.39), weak (0.40–0.59), moderate (0.60–0.79), strong (0.8–0.90), or almost perfect (>0.90) agreement was accepted for kappa coefficients categories (McHugh et al. 2012). Changes in P4 concentrations over time were analysed by a paired‐samples *t*‐test. P4 values were compared between pregnant and nonpregnant groups (based on day 42 TAUS) using the Mann–Whitney U test due to the normality was not hold. Statistical significance was set at *p* < 0.05 for all analyses. Data normality was evaluated using skewness and kurtosis values (±1.5). AOFPT characteristics (sensitivity, specificity, positive predictive value and negative predictive value) were calculated according to Kastelic 2006. The accuracy of blood serum progesteron level in predicting the presence of the pregnancy were estimated by receiver operating characteristic (ROC) analysis by calculating area under the curve (AUC) and 95% confidence intervals. Low (0.5< AUC ≤0.7), moderate (0.7< AUC ≤0.9), or high (0.9< AUC ≤1) accuracy was accepted for AUC categories (Gardner and Greiner 2006). All analyses were performed using SPSS Statistics for Windows, version 26.0 (International Business Machines Corporation‐IBM, Armonk, NY, USA).

## 3 Results

One of the goats in the study expired. Table 1 presents the results of the pregnancy‐associated glycoprotein test (AOFPT) on Days 21 and 28, the serum progesterone concentration (P4) on Days 21 and 28, and the transabdominal ultrasonography (TAUS) on Days 35 and 42. According to AOFPT findings, 14 false negatives and 1 false positive were determined on Day 21, and 3 false positives on day 28. According to P4 findings, 5 false positives were detected on Day 21 and 6 false positives on Day 28. In the TAUS examination on Day 35, although gestational sacs were observed in two goats, the absence of embryonic movement or heartbeat, which are necessary for a positive pregnancy diagnosis, resulted in a suspected diagnosis for these two goats on that day. On Day 42 TAUS examination, these two goats were diagnosed as nonpregnant due to lack of the pregnancy criteria aforementioned before.

**TABLE 1:** Goats' pregnancy‐related findings following the application of the examination techniques.

	AOFPT		P4		TAUS	
	Day 21	Day 28	Day 21	Day 28	Day 35	Day 42
	*n*	*n*	*n*	*n*	*n*	*n*
**Pregnant**	54	68	68	68	68	68
**Non‐pregnant**	15	13	11	10	14	16
**False positive**	1	3	5	6	—	—
**False negative**	14	—	—		—	—
**Suspect**	—	—	—		2	—
**Total**	84	84	84	84	84	84

Abbreviations: AOFPT: The Alertys OnFarm Pregnancy Test; P4: serum progesterone; TAUS: transabdominal ultrasonography.

The pregnancy rate was found to be 81.0% based on the results of the 42nd day of the TAUS examination, which was approved as the study's gold standard. The AOFPT with the study's gold standard examination findings results on Day 21 was examined, and the kappa value was calculated as 0.55 at a 95% confidence interval. For the Day 21 AOFPT, the sensitivity, specificity, positive predictive value, negative predictive value, and accuracy rate were determined as 79.41%, 93.75%, 98.18%, 51.72% and 82.14%, respectively (Table 2). The AOFPT results on Day 28 was the kappa value was calculated as 0.87 at a 95% confidence interval. For the Day 28 AOFPT, the sensitivity, specificity, positive predictive value, negative predictive value and accuracy rate were determined as 100%, 81.25%, 95.77%, 100.00% and 96.43%, respectively (Table 3).

**TABLE 2:** Consistency of pregnancy‐associated glycoprotein test on Day 21.

		Day 42 TAUS				
		Pregnancy (‐)		Pregnancy (+)		
		*n*	%	*n*	%	Total
**Day 21 AOFPT**	**Pregnancy (‐)**	15	93.8	14	20.6	29
	**Pregnancy (+)**	1	6.3	54	79.4	55
	**Total**	16		68		84

Kappa = 0.55, sem = 0.10, CI = 0.37–0.74 Sensitivity = 79.41%, Specificity = 93.75%, Positive Predictive Value = 98.18, Negative Predictive Value = 51.72, Accuracy Rate = 82.14%.

**TABLE 3:** Consistency of pregnancy‐associated glycoprotein test on Day 28.

		Day 42 TAUS				Total
		Pregnancy (‐)		Pregnancy (+)		
		*n*	%	*n*	%	
**Day 28 AOFPT**	**Pregnancy (‐)**	13	81.3	0	0.0	13
	**Pregnancy (+)**	3	18.8	68	100.0	71
	**Total**	16		68		84

Kappa = 0.87, sem = 0.07, CI = 0.74–1.00 Sensitivity = 100.00%, Specificity = 81.25%, Positive Predictive Value = 95.77, Negative Predictive Value = 100.00, Accuracy Rate = 96.43%.

A comparison of serum progesterone concentrations on Day 21 and Day 28 according to the reference method examination findings is presented in Table 4. The results of the 42nd TAUS indicated that pregnant goats exhibited significantly higher P4 values (median ± IQR) than non‐pregnant goats on the 21st (5.95 ± 2.60 vs. 0.14 ± 2.73) and 28th day (6.92 ± 2.93 vs. 0.84 ± 6.06) (*p* < 0.001).

**TABLE 4:** Comparison of progesterone concentrations (ng/mL) according to reference method findings.

	Day 42 TAUS						
	Pregnancy (‐)			Pregnancy (+)			
	Med	Q1	Q3	Med	Q1	Q3	*p*
**Day 21 P4**	0.143	0.112	2.840	5.945	4.665	7.260	<0.001
**Day 28 P4**	0.837	.070	6.130	6.915	6.035	8.965	<0.001

Abbreviations: Med = median, Mann–Whitney *U* test, Q1: first quartile (25%), Q3: third quartile (75%).

**FIGURE 1:**
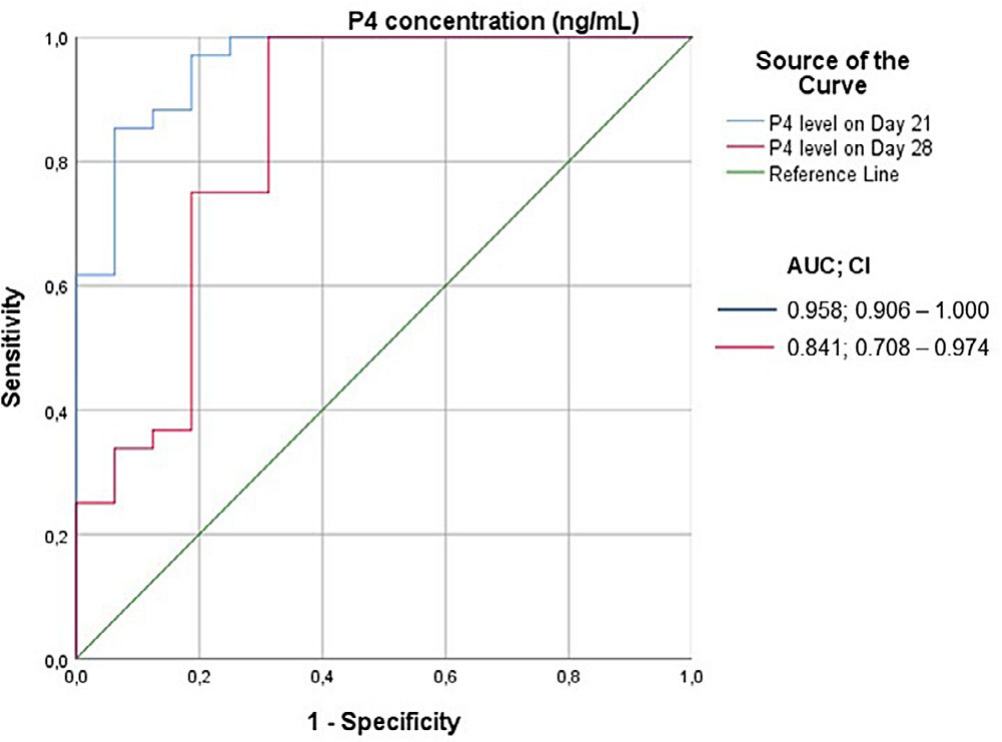
ROC curve of progesterone concentrations according to reference method findings.

According to the gold standard examination findings, serum progesterone concentration analysed on day 21, the AUC value was found to be 0.958, indicating high accuracy (Figure 1 and Table 5). The cutoff value was determined to be >4.19 ng/mL. Sensitivity was 88.2%, and specificity was 87.5% (Table 5). On Day 28, the AUC value was found to be 0.841 (Figure 1 and Table 5), which was lower than that on Day 21. The cut‐off value was determined to be >6.13 ng/mL. Sensitivity was 75%, and specificity was 75% (Table 5).

**TABLE 5:** Ideal cut‐off values of progesterone concentrations.

	AUC	CI		Cut‐off value of P4 (ng/mL)	*p*	Sensitivity (%)	Specificity (%)
		LL	UL				
**Day 21 P4**	0.958	0.906	1.000	4.19	0.000	88.2	87.5
**Day 28 P4**	0.841	0.708	0.974	6.13	0.000	75	75

Abbreviations: AUC: area under the process characteristic (ROC; receiver operating characteristic) curve, CI: confidence interval, LL: lower limit, UL: upper limit.

AOFPT‐identified pregnant goats exhibited significantly higher P4 levels (*p* < 0.001) than non‐pregnant goats on Day 21 (5.98 ± 3.21 vs. 4.05 ± 5.46) and Day 28 (6.84 ± 3.06 vs. 0.75 ± 5.96) (Table 6).

**TABLE 6:** Comparison of the P4 concentration (ng/mL) based on the 21st and 28th day test results.

	Day 21 and 28 AOFPT						
	Pregnancy (‐)			Pregnancy (+)			
	Med	Q1	Q3	Med	Q1	Q3	*p*
**Day 21 P4**	4.050	0.130	5.590	5.980	4.530	7.7390	<0.001
**Day 28 P4**	0.754	0.082	6.040	6.840	5.770	8.830	<0.001

Abbreviations: Med = median, Mann–Whitney *U* test, Q1: first quartile (25%), Q3: third quartile (75%).

## 4 Discussion

The present study aimed to evaluate and validate the performance of the AOFPT, a PAG‐based lateral flow test, for early pregnancy diagnosis in goats on Days 21 and 28 post‐mating. In the present study, even though statistical significance was detected (*p* < 0.001), the agreement between Day 21 AOFPT results and the reference test yielded a Kappa value of 0.55 (95% CI: 0.37–0.74), indicating weak agreement. The sensitivity of AOFPT on Day 21 was 79.41%, specificity was 93.75%, PPV was 98.18%, and NPV was 51.72% (Table 2). On the other hands, for Day 28, the Kappa value between AOFPT and the reference method was 0.87 (95% CI: 0.74–1.00), indicating strong agreement (*p* < 0.001). The sensitivity of AOFPT was 100%, specificity was 81.25%, PPV was 95.77%, and NPV was 100% (Table 3). These results demonstrate that the test correctly identified pregnancy in 95.77% of does that were pregnant and correctly excluded pregnancy in 100% of non‐pregnant does. Akköse (2023) noted that timely identification of nonpregnant cows facilitates earlier re‐insemination and emphasized that the primary goal of pregnancy diagnosis should be accurate detection of nonpregnant animals rather than simply identifying pregnant ones. In this context, Day 28 AOFPT results in the present study were highly successful in correctly identifying both pregnant and nonpregnant does.

In studies conducted by Akköse (2023) to determine the diagnostic accuracy of two commercial PAG tests (IDEXX On‐Farm Pregnancy Test [OFPT] and IDEXX Rapid Visual Pregnancy Test [RVPT]) for early pregnancy detection in dairy cows at Days 28–31 post‐insemination, OFPT demonstrated sensitivity, specificity, PPV, and NPV of 100%, 93.1%, 89.1% and 100%, respectively, whereas the RVPT demonstrated sensitivity, specificity, PPV, and NPV of 97.4%, 92.1%, 87.4% and 98.4%, respectively. Both tests exhibited excellent discrimination between pregnant and nonpregnant cows (both AUCs > 0.90). Szelenyi et al. (2024) reported sensitivity, specificity, PPV, and NPV values of 98.9%, 88.7%, 86.8% and 99.1%, and overall accuracy of 93.1% for OFPT on Days 28–35 post‐insemination. In heifers, the test showed 100% sensitivity and 81.6% specificity; in multiparous cows, sensitivity and specificity were 98.5% and 89.5%, respectively. Kline et al. (2024) validated OFPT by comparing the accuracy of three commercial PAG assays with transrectal ultrasonography; they reported a significant positive correlation between OFPT and transrectal ultrasonography (*r* = 0.77; *p* < 0.01) and a concordance rate of 92.4% between the two methods. In another study conducted in Awassi ewes also indicated that the test could be considered a practical, rapid, and reliable method for early pregnancy detection (sensitivity 95.55 %, accuracy 93.00 %, and kappa = 0.859) starting from the 28th day after mating (Uyanık et al. 2026). In the present study, AOFPT performance on both days in goats was comparable to results reported in cattle and ewes, confirming its suitability for early pregnancy diagnosis.

In sheep and goats, PAG concentrations become detectable as early as Days 17–18 post‐breeding and reach 3–5 ng/mL around Days 21–22 (Sousa et al. 1999). Salve et al. (2016) reported that in goats, PAG concentrations increased significantly (*p* < 0.01) from Day 16 of pregnancy through Days 20, 24 and 28. Zamfirescu et al. (2011) stated that PAG concentrations begin to increase from Day 16 post natural mating. Detection of the antigen and markedly elevated concentrations between Days 16 and 20 in goats coincides with the time when the trophoblast firmly adheres to the uterine wall and placentation begins. Therefore, the presence of PAGs in the dam's serum not only serves as a useful pregnancy diagnostic tool between Days 21 and 24 post‐conception but also provides information on embryonic and/or foetal viability (Gonzalez et al. 2004). The presence of 14 false‐negative AOFPT results on Day 21 (Table 1) in the present study may be explained by the gradually increasing PAG concentrations reported by Salve et al. (2016) between Days 20 and 28. Additionally, as noted in the same study, some goats may have had PAG concentrations below the detectable threshold, which could also account for 14 false‐negative results in the test outcomes.

P4 plays a critical role in implantation, embryonic development, and maintenance of pregnancy in mammals. In goats, the ovaries are the primary source of P4 throughout both the estrous cycle and pregnancy (Charallah et al. 2010; Sharma et al. 2020a). Serum P4 concentrations increase gradually as pregnancy progresses, then decline to baseline levels at the onset of parturition, abortion, or embryonic death (Barik and Yadav 2022). Elevated P4 levels (above 1 ng/mL) indicate the presence of a functional corpus luteum and are generally associated with pregnancy or the diestrus phase of the cycle; however, they may also be observed under abnormal conditions, such as extended estrous cycles, early embryonic death, luteal cysts, or pseudopregnancy (Cruz et al. 2024). Medan et al. (2004) reported a mean P4 concentration of 7.4 ± 0.5 ng/mL in pregnant goats on Day 21 with no statistical difference in P4 concentrations between pregnant and nonpregnant goats on Day 7 and found that on Day 21, P4 concentrations correlated with ultrasonographic findings, yielding accuracy rates of 80% for pregnant and 100% for nonpregnant goats. Gonzalez et al. (2004) reported that all pregnant goats had mean P4 levels of 8.42 ± 0.23 ng/ml on Day 22, whereas 22 nonpregnant goats (34.4%) exhibited P4 concentrations similar to pregnant goats (8.01 ± 0.75 ng/mL), resulting in a 34.4% false‐positive rate. Among those 22 nonpregnant goats, five had basal P4 levels on Day 26, whereas the remaining 17 maintained functional P4 levels (7.72 ± 0.83 ng/mL) on Day 26. The authors concluded that this may have been associated with early embryonic death, hydrometra, or luteal cysts that prolong the lifespan of the corpus luteum. Salve et al. (2016) reported that in goats, P4 concentrations increased significantly by day 8 post‐insemination and remained relatively stable until Day 12 of pregnancy, after which they increased significantly from Day 16 (3.76 ± 0.15 ng/mL) to Day 20 (6.25 ± 0.33 ng/mL), Day 24 (8.38 ± 0.26 ng/mL) and Day 28 (11.39 ± 0.40 ng/mL) (*p* < 0.01). Cruz et al. (2024) reported P4 concentration of 8.15 ± 1.05 ng/mL in pregnant goats on Day 28. In the present study, mean P4 concentrations in pregnant goats, based on the reference method, were 6.30 ± 0.27 ng/mL on Day 21 and 7.50 ± 0.28 ng/mL on Day 28. These results are consistent with those of Cruz et al. (2024), Gonzalez et al. (2004), Medan et al. (2004) and Salve et al. (2016). Serum P4 values in pregnant goats were significantly higher than those in nonpregnant goats on Days 21 and 28 (*p* < 0.001) (Table 4). The P4 cut‐off concentrations for pregnancy prediction were determined to be >4.19 ng/mL on Day 21 and >6.13 ng/mL on Day 28 (Table 5). The Day 21 P4 cut‐off provided higher specificity and sensitivity for predicting pregnancy (Figure 1).

When non‐pregnant goats (based on the gold standard method) were evaluated individually, of the 16 non‐pregnant does, seven had P4 concentrations of <1.5 ng/mL on Day 21 and remained <1.5 ng/mL on Day 28; four had P4 levels of >1.5 ng/mL on Day 28; three had P4 concentrations of >1.5 ng/mL on Day 21 but returned to basal levels by Day 28; and two maintained P4 concentrations of >1.5 ng/mL on Day 28. In the first seven does, estrous cyclicity may not have been active; in four does, they may have been in the diestrus phase of a new estrous cycle; and in the remaining five, measurement error or other conditions, such as early embryonic death, hydrometra, or luteal cysts—which can extend the lifespan of the corpus luteum—may have been present, as noted by Gonzalez et al. (2004). Among these five does, one produced false‐positive AOFPT results on both Days 21 and 28, whereas the others produced false‐positive results only on Day 28. These data suggest that embryonic death may have occurred in these animals. Previous studies have reported that the half‐life of PAGs is approximately 7.5–8 days (Haugejorden et al. 2006; Mialon et al. 1993). Following embryonic death, a delayed decrease or persistence of high PAG concentrations may result in false‐positive AOFPT outcomes.

Ultrasonography is a valuable diagnostic imaging technique used in reproductive management of both small and large ruminants (Jones and Reed 2017; Wojtasiak et al. 2023). In small ruminants, anechoic fluid within the uterine lumen can be detected transabdominally on Days 25–28 (Devi et al. 2019). However, the presence of fluid in the uterine lumen should not constitute the sole basis for confirming pregnancy; definitive diagnosis requires visualization of the amniotic vesicle and detection of the foetal heartbeat (Roberts 2022). In goats, foetal heartbeats can be detected between Days 27 and 35 using a transabdominal approach (Suguna et al. 2008). Placentomes also become visible via transabdominal ultrasonography around Day 40 of gestation (Erdoğan 2012). Tekin and Köse (2022) reported that TAUS performed on Days 35 and 50 showed high concordance in ewes (κ = 0.864, *p* < 0.001), and TAUS performed on Day 35 accurately determined pregnancy status 93.93% of the time. Jones et al. (2016) reported that the transabdominal method had a sensitivity of 40% on Day 21 and 100% on Day 39 of pregnancy. Sensitivity increases after Day 40, when the uterus assumes an intraabdominal position. Therefore, the ideal time for pregnancy diagnosis via TAUS is considered to be between Days 40 and 75 (Fthenakis et al. 2012; Lone et al. 2016; Mali et al. 2022). In the study TAUS was performed twice on Days 35 and 42, and Day 42 was considered the gold standard. In the present study, two does were provisionally diagnosed as suspicious at the first examination; during the second examination, these suspicious diagnoses were resolved, and the does were determined to be nonpregnant (Table 1). Individual retrospective records showed that both of these does had P4 concentrations of >1.5 ng/mL on Day 28 and yielded false‐positive AOFPT results on Day 28. It was assessed that embryonic loss may have occurred in these animals.

Early pregnancy detection and determination of offspring number are critical for improving reproductive efficiency in goats. Accurate pregnancy diagnosis provides essential information for effective herd management practices (Suguna et al. 2008). TAUS is effective for early pregnancy detection in goats; however, it requires training and expertise. On a herd‐by‐herd basis, this method may be less accessible for producers with limited resources or for large‐scale dairy operations, as ultrasonographic examination of many animals demands significant time, labour, skilled personnel and expensive equipment. In such cases, serological methods may prove more practical and efficient than ultrasonography (Lv et al. 2021). Nonetheless, relying solely on circulating P4 concentrations to monitor pregnancy in goats can lead to misdiagnoses, because P4 levels may be influenced by pathophysiological conditions, such as luteal cysts, pseudopregnancy, or early embryonic loss (Charallah et al. 2010).

In this context, serological tests that detect circulating PAGs, which are markers of fetoplacental unit presence, serve as useful diagnostic tools for early pregnancy detection under typical farm management conditions (Cruz et al. 2024). The advantage of these tests is that they provide rapid results, do not require trained personnel or expensive equipment, and can be applied even in field conditions (Rial et al. 2024). A limitation of the present study is that although AOFPT is simple, rapid, and does not require specialized labour (it provides on‐site results immediately after blood collection), it is a qualitative assay rather than a quantitative one. Consequently, it cannot measure PAG concentrations precisely or provide information on foetal viability or litter size.

In conclusion, the commercial AOFPT developed to detect PAG molecules in bovine serum, was observed to be a rapid, practical, and effective method for early pregnancy diagnosis for to improve reproductive performance and herd management strategies in goats, especially following the post‐mating 28th day. However, if the results are false negative or false positive, it is recommended that the test be repeated or the animal be re‐examined by using the gold‐standard TAUS test. Moreover, according to the authors' knowledge, this study seems to be the initial comparative evaluation of serum P4 measurement and the OnFarm Pregnancy Test versus TAUS for early pregnancy diagnosis in goats.

## Author Contributions

**Ayşe Merve Köse**: conceptualization, methodology, investigation, writing (original draft, review and editing). **Mehmet Can Kara**: methodology, investigation, formal analysis, writing (original draft).

## Funding

The authors have nothing to report.

## Ethical Statement

This study was conducted with the approval of the Hatay Mustafa Kemal University Rectorate Animal Experiments Local Ethics Committee's decision numbered 2022/05‐02.

## Conflicts of Interest

The authors declare no conflicts of interests.

## Data Availability

The data may be provided on reasonable request.
